# Predicting early Alzheimer’s with blood biomarkers and clinical features

**DOI:** 10.1038/s41598-024-56489-1

**Published:** 2024-03-13

**Authors:** Muaath Ebrahim AlMansoori, Sherlyn Jemimah, Ferial Abuhantash, Aamna AlShehhi

**Affiliations:** 1 Department of Biomedical Engineering, Khalifa University, P.O. Box: 127788, Abu Dhabi, United Arab Emirates; 2 Healthcare Engineering Innovation Center (HEIC), Khalifa University, P.O. Box: 127788, Abu Dhabi, United Arab Emirates

**Keywords:** Alzheimer’s disease, Machine learning, Blood biomarkers, Clinical features, Computational biology and bioinformatics, Computational neuroscience, Data integration, Data mining, Machine learning, Microarrays, Neurological disorders, Engineering, Biomedical engineering, Computational biology and bioinformatics, Computational neuroscience, Data integration, Data mining, Machine learning, Microarrays, Neurological disorders, Engineering, Biomedical engineering

## Abstract

Alzheimer’s disease (AD) is an incurable neurodegenerative disorder that leads to dementia. This study employs explainable machine learning models to detect dementia cases using blood gene expression, single nucleotide polymorphisms (SNPs), and clinical data from Alzheimer’s Disease Neuroimaging Initiative (ADNI). Analyzing 623 ADNI participants, we found that the Support Vector Machine classifier with Mutual Information (MI) feature selection, trained on all three data modalities, achieved exceptional performance (accuracy = 0.95, AUC = 0.94). When using gene expression and SNP data separately, we achieved very good performance (AUC = 0.65, AUC = 0.63, respectively). Using SHapley Additive exPlanations (SHAP), we identified significant features, potentially serving as AD biomarkers. Notably, genetic-based biomarkers linked to axon myelination and synaptic vesicle membrane formation could aid early AD detection. In summary, this genetic-based biomarker approach, integrating machine learning and SHAP, shows promise for precise AD diagnosis, biomarker discovery, and offers novel insights for understanding and treating the disease. This approach addresses the challenges of accurate AD diagnosis, which is crucial given the complexities associated with the disease and the need for non-invasive diagnostic methods.

## Introduction

Alzheimer’s disease (AD) is a complex neurodegenerative disorder characterized by a gradual loss of memory and cognition^1,2^. AD is the most common cause of dementia, and is projected to affect over 78 million people by 2030^3^. Although the exact cause of AD is unknown, its hallmark is the buildup of abnormal protein deposits in the brain known as amyloid plaques and tau tangles. With no cure, early detection is crucial to allow timely interventions^4,5^. Currently, the detection of conventional AD biomarkers of amyloid-$\beta$ deposition and tau pathology require expensive or invasive diagnostic tools, such as Positron Emission Tomography (PET), Magnetic Resonance Imaging (MRI), and cerebrospinal fluid (CSF) sampling^6,7^. These procedures are not conducted routinely, precluding early diagnosis. Additionally, these biomarkers are present in other forms of dementia^8,9^ and cognitively normal (CN) individuals^10,11^, hindering definitive diagnosis. Several studies have also shown that these AD biomarkers perform poorly in distinguishing between the early and late stages of AD. A meta-analysis of CSF tau levels in identifying mild cognitive impairment (MCI) cases that progress to AD showed wide variation in specificity, ranging from 0.48 to 0.72^12^. Another meta-analysis of $\beta$-amyloid PET showed poor specificity in differentiating between MCI and AD patients^13^. Cumulatively, these factors create a significant potential for misdiagnosis.

Due to the drawbacks of conventional AD biomarkers, blood-based biomarkers have been proposed for Alzheimer’s disease diagnosis. The use of blood biomarkers for the diagnosis of AD was made possible recently by the development of novel, high-sensitivity assays. Blood concentrations of amyloid-$\beta$ and phosphorylated tau appear to correlate with their corresponding levels in CSF^14^. A study examined the predictive power of plasma biomarkers, such as APP669-711/A$\beta$1-42 and A$\beta$1-40/A$\beta$1-42, and their combinations, in identifying patients with positive or negative brain amyloid-$\beta$ status. Two distinct datasets, one from Japan (n = 121) and one from Australia (n = 252), were used to examine cognitive states using amyloid-$\beta$-PET imaging. Researchers found that plasma biomarkers accurately predict brain amyloid-$\beta$ load, suggesting their potential for cost-effective and scalable population screening^15^. As a matter of fact, a plasma test utilizing mass spectrometry analysis of A$\beta$ has received approval in accordance with the Clinical Laboratory Improvement Amendments (CLIA) for the purpose of detecting A$\beta$ pathology^16^. In another clinical trial , lecanemab (BAN2401), an IgG1 monoclonal antibody, was tested for targeting soluble amyloid beta (A$\beta$) in various forms and the Clinical Dementia Rating-Sum-of-Boxes (CDR-SB) is used as one of the key secondary endpoints. The trial evaluated three doses and two regimens of lecanemab to placebo in early Alzheimer’s disease, mild cognitive impairment, and mild dementia using a Bayesian design with response-adaptive randomization. The 18-month analyses showed brain amyloid reductions and clinical improvements, suggesting therapeutic benefits. According to Bayesian and frequentist studies, the Clinical Dementia Rating-Sum-of-Boxes (CDR-SB) decreased 33% and 26% from placebo^17^. Further monitoring of protein levels in the brain is done through plasma levels of mitochondrial proteins from neuronal-derived exosomes (NDEs)^18^. Other blood markers, such as neurofilament light chain and glial fibrilary acidic protein may indicate Alzheimer’s disease progression and facilitate monitoring of treatment effects^14^. The significance of these blood-based biomarkers is reinforced by evidence of systemic changes in blood cells that reflect Alzheimer’s disease pathology in the brain^19,20^.

Using big data sets to find patterns and associations, artificial intelligence (AI) and machine learning (ML) techniques have demonstrated potential in the analysis of blood biomarkers for Alzheimer’s disease diagnosis^21^. Lee and Lee^22^ tested several ML techniques, such as Support Vector Machines (SVM) and Random Forest (RF), to distinguish between cognitively normal (CN) and AD participants. The input data consisted of blood gene expression data from the AddNeuroMed^23^ and Alzheimer’s Disease Neuroimaging Initiative (ADNI)^24^ cohorts. Their SVM model achieved an AUC (area under receiver operator curve) of 0.62 using ADNI as the test dataset. Oriol et al.^25^ utilized Bootstrap Stage-Wise Model Selection (BSWiMS), Least Absolute Shrinkage and Selection Operator (LASSO), Recursive Partitioning and Regression Trees (RPART), and a BSWMS-LASSO-RPART ensemble to differentiate between AD and CN participants from ADNI using blood-derived genetic variation data. They showed that the ensemble method has better performance with an AUC of 0.72. An XGBoost model utilizing plasma metabolites achieved an AUC of 0.89 in the detection of AD and CN cases^26^. Logistic regression using plasma levels of inflammatory proteins enabled the differentiation of AD from controls (AUC 0.79) and MCI subjects (AUC 0.74)^27^.

Multimodal machine learning models, (i.e., models incorporating multiple types of input), have been proposed to improve diagnostic accuracy over single biomarkers such as A$\beta$ PET^13^. Some machine learning models have achieved enhanced performance in AD prediction by combining their primary input data with clinical features^28–31^. An RF model using MRI and demographic data from a small cohort of 49 subjects in the Vienna Trans-Danube Aging study attained an AUC of 0.77 in predicting whether neuropathological changes are present^28^. Zhu et al.^32^ used MRI data and the APOE4 genotype in a wide neural network to predict cognitive decline in A$\beta$-positive individuals with an accuracy of 0.86. An ensemble of logistic regression, support vector machine, and gradient boosting methods achieved an AUC of 0.87 for early diagnosis of cognitive impairment using demographic and MRI data from the Epidemiology of Dementia in Singapore study^30^. A neural network for predicting MCI diagnosis using radiomic features and amyloid brain PET attained an AUC of 0.90 using 656 subjects from ADNI and a EudraCT (European Union Drug Regulating Authorities Clinical Trials Database) cohort^31^. An RF model of serum biomarker data and clinical features attained an AUC of 0.94 in distinguishing between CN and AD cases^33^. However, despite the progress made in AI-based detection of AD, most ML models suffer from a black-box reputation among clinicians^34^.

In this study, we present a machine learning approach to accurately predict MCI/AD and identify novel blood-based biomarkers. We developed a multimodal ML method to distinguish between CN and MCI/AD cases. SHapley Additive exPlanation (SHAP)^35^ was used to identify clinical and genetic features that can serve as potential biomarkers. To develop our model, various ML methods were evaluated, namely Support Vector Machines (SVM), AdaBoost, Random Forest (RF), and Multilayer Perceptron (MLP), in combination with different feature selection methods: Least Absolute Shrinkage and Selection Operator (LASSO), Chi-square, mutual information (MI) and none. The performance of each model was tested with combinations of genotyping, gene expression, and clinical data. Our study demonstrates that multi-modal data leads to improved performance compared to single-modality data, while also highlighting that single-modality data prompts the model to emphasize the top features within that specific data modality. To address the black-box nature of ML models, SHAP is used to enable a better understanding of the model’s decision-making process by offering insights into how various features or variables contribute to the model’s output. This significantly contributes in aiding clinicians in making wise judgments and strengthens diagnostic abilities by enhancing interpretability, validating predictions, and identifying previously undiscovered biomarkers.

## Results

A machine learning workflow with various feature selection techniques, models, and hyperparameter tuning was developed to identify the best-performing ML method and the best features to predict MCI/AD. SNPs (single nucleotide polymorphisms), gene expression, and clinical data were preprocessed and combined in different ways to make the multimodal data inputs. Feature selection was performed using Chi-square, Mutual Information (MI), and LASSO techniques. The selected features were used to train binary classification models, including SVM, RF, AdaBoost, and MLP. Hyperparameter optimization was performed for each model and input data combination to obtain the highest possible performance. Figure 5 in the “Methods” section shows the model development workflow. The results are explained in detail in the following sections.

### Study cohort characteristics

Table 1 summarizes the demographic and clinical characteristics of the participants in the CN and MCI/AD groups. There is a significant differences in the mean age of the CN (74.6 ± 5.4 years) and MCI/AD (72.7 ± 7.6 years) groups (p = 0.001) was observed from the clinical data. The genders distribution is relatively balanced in CN participants (107 males to 105 females), but unbalanced in MCI/AD (162 females to 252 males). As expected, there are significant differences in most clinical features between the CN and MCI/AD groups.

**Table 1:** Study cohort statistics and clinical features summary in CN and MCI/AD groups.

	CN	AD/MCI	P-value
Number of patients (total = 623)	212	411	
Age	74.62 ± 5.44	72.69 ± 7.57	0.001
Gender (male)	107 (50.5)	251 (60.8)	0.017
APOE4 allele	58	231	< 0.001
Years of education	16.23 ± 2.67	15.97 ± 2.79	0.258
FDG	1.30 ± 0.11	1.22 ± 0.16	< 0.001
AV45	1.10 ± 0.18	1.22 ± 0.24	< 0.001
ABETA	1241.52 ± 429.99	1017.68 ± 450.14	< 0.001
TAU	247.91 ± 81.60	283.65 ± 131.37	0.005
PTAU	22.60 ± 8.35	27.04 ± 14.47	0.001
CDRSB	0.07 ± 0.30	2.50 ± 2.53	< 0.001
ADAS11	5.82 ± 2.84	12.03 ± 8.13	< 0.001
ADAS13	9.33 ± 4.32	18.63 ± 11.21	< 0.001
ADASQ4	2.94 ± 1.67	5.63 ± 2.88	< 0.001
MMSE	29.06 ± 1.24	26.41 ± 4.02	< 0.001
RAVLT_immediate	45.34 ± 10.55	33.46 ± 12.41	< 0.001
RAVLT_learning	5.72 ± 2.32	4.13 ± 2.76	< 0.001
RAVLT_forgetting	4.00 ± 2.85	4.46 ± 2.57	0.043
RAVLT_perc_forgetting	37.46 ± 27.34	61.10 ± 40.21	< 0.001
LDELTOTAL	14.11 ± 3.46	6.48 ± 4.38	< 0.001
TRABSCOR	82.18 ± 37.88	124.56 ± 72.44	< 0.001
FAQ	0.24 ± 1.07	6.05 ± 7.88	< 0.001
MOCA	25.46 ± 2.45	21.85 ± 4.73	< 0.001
EcogPtMem	1.54 ± 0.44	2.27 ± 0.71	< 0.001
EcogPtLang	1.38 ± 0.38	1.83 ± 0.63	< 0.001
EcogPtVisspat	1.13 ± 0.23	1.45 ± 0.58	< 0.001
EcogPtPlan	1.14 ± 0.25	1.52 ± 0.60	< 0.001
EcogPtOrgan	1.29 ± 0.40	1.67 ± 0.72	< 0.001
EcogPtDivatt	1.45 ± 0.51	1.92 ± 0.78	< 0.001
EcogPtTotal	1.33 ± 0.30	1.80 ± 0.56	< 0.001
EcogSPMem	1.27 ± 0.35	2.46 ± 0.94	< 0.001
EcogSPLang	1.13 ± 0.22	1.92 ± 0.83	< 0.001
EcogSPVisspat	1.07 ± 0.19	1.71 ± 0.83	< 0.001
EcogSPPlan	1.13 ± 0.23	1.90 ± 0.92	< 0.001
EcogSPOrgan	1.14 ± 0.36	2.01 ± 1.00	< 0.001
EcogSPDivatt	1.22 ± 0.39	2.23 ± 0.98	< 0.001
EcogSPTotal	1.16 ± 0.22	2.04 ± 0.82	< 0.001
Ventricles	34,517.16 ± 18,550.93	41,918.44 ± 24,315.44	< 0.001
Hippocampus	7270.30 ± 956.41	6743.25 ± 1263.40	< 0.001
WholeBrain	1,018,671.18 ± 107,104.07	1,033,167.05 ± 117693.19	0.141
Entorhinal	3754.57 ± 666.69	3491.00 ± 825.96	< 0.001
Fusiform	18,144.35 ± 2461.02	17,795.75 ± 2981.73	0.218
MidTemp	19,974.68 ± 2681.84	19,767.19 ± 3232.69	0.499
ICV	1,500,236.24 ± 156,407.97	1,533,944.09 ± 159,949.57	0.015
mPACCdigit	0.12 ± 2.94	− 8.16 ± 7.87	< 0.001
mPACCtrailsB	0.05 ± 2.66	− 7.22 ± 7.21	< 0.001

Continuous variables are expressed as mean ± standard deviation.

CN: cognitively norma; AD: Alzheimer’s disease; MCI: mild cognitive impairment. More details about the clinical features can be found in the “Alzheimer’s Disease Neuroimaging Initiative” section under “Methods”.

### Performance evaluation of ML models

The machine learning models’ performance was assessed across four distinct input scenarios: clinical data with cognitive scores, clinical data without cognitive scores, gene expression data, and SNP data. Furthermore, various combinations of these four types of data were also considered in the evaluation process. The clinical data is used in two ways because incorporating cognitive scores (CS) aligns with diagnostic criteria, providing a straightforward and clinically relevant approach that enhances the model’s sensitivity to early AD cognitive impairments. On the other hand, excluding cognitive scores forces the model to use other biomarkers, which may reveal novel predictive features. This shift may lead to the utilization of fewer intuitive biomarkers, potentially reducing the model’s clinical interpretability. Four feature selection methods were employed, namely Chi-square, mutual information (MI), Least Absolute Shrinkage and Selection Operator (LASSO), and no feature selection, for each input. The results of prediction models for all combinations of inputs are detailed in Supplementary Table S1.

**Figure 1:**
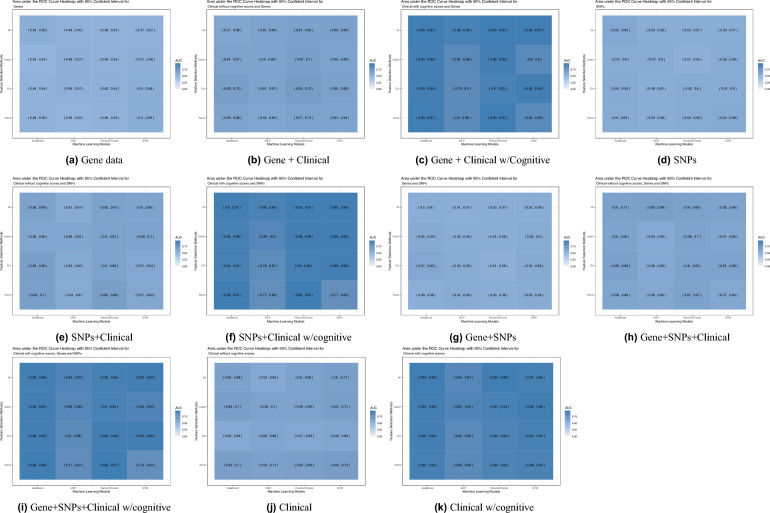
Heatmap showing area under the ROC Curve (AUC) with 95% confident interval of ML models for all data inputs: gene expression, SNPs, Clinical features without cognitive scores, and Clinical features with cognitive scores. SVM: Support Vector Machines; MLP: multilayer perceptron.

**Figure 2:**
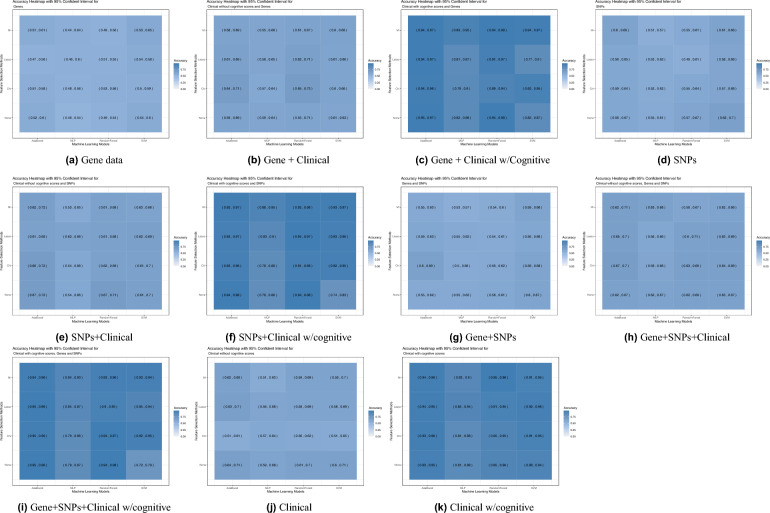
Heatmap showing the accuracy with 95% confident interval of ML models for all data inputs: gene expression, SNPs, Clinical features without cognitive scores, and Clinical features with cognitive scores. SVM: Support Vector Machines; MLP: multilayer perceptron.

To start, the evaluation of the model’s performance focused on gene expression data, both independently and in combination with clinical data, excluding cognitive scores (CS). For gene expression with clinical without CS input, the Random Forest classifier with Chi-square feature selection outperformed all other models with an AUC of 0.65 and an accuracy of 0.65 as can be seen in Figs. 1b, 2b. All ML models performed well achieving an AUC of 0.57 or higher, with accuracy ranging from 0.55 for MLP to 0.65 for the Random Forest classifier. For gene expression data only the models’ performance showed a decline where the best performing model was also the Random Forest classifier with Chi-square feature selection achieving an AUC of 0.52 and an accuracy of 0.53 as can be seen in Figs. 1a, 2a.

Next, the performance evaluation concentrated on SNP data alone and in conjunction with clinical information, with an intentional exclusion of cognitive scores (CS). Using SNPs and clinical data without CS, the best performing model was the Random Forest with no feature selection with an AUC of 0.63 with upper bound of 0.7 and an accuracy of 0.67 (Figs. 1e, 2e). Using SNP data alone the performance exhibited an overall decrease across the models, as was the case with gene data, with the best performing model being the Adaboost with MI feature selection giving an AUC of 0.55 and an accuracy of 0.60 (Figs. 1d, 2d). Subsequently, various combinations of gene expression, SNP, and clinical data without CS were employed as inputs to assess the models’ performance. The combination of gene and SNPs yielded with the SVM model without feature selection as the best performing model an AUC of 0.53 and an accuracy of 0.60 (Figs. 1g, 2g), which is higher than using gene expression alone. Adding clinical data without CS to this combination increased the performance with the best performing model being MLP with MI feature selection to an AUC of 0.63 and an accuracy of also 0.63 (Figs. 1h, 2h).

Lastly, the performance was evaluated for the combinations of clinical with cognitive scores, gene expression, and SNP data. Substantial performance improvements were observed in input data combinations containing clinical data with cognitive scores. When clinical data is used as the sole input, the SVM model without feature selection achieves the highest performance, as evidenced by an accuracy of 0.6 and an AUC of 0.63, as shown in Figs. 1j, 2j. The substantial performance mentioned before is adamant when cognitive scores are added to the set of clinical features to reach an AUC = 0.94 and accuracy = 0.95 using Random Forest classifier and MI feature selection as seen in Figs. 1k, 2k. In the evaluation of multiple feature inputs, the clinical data with CS was combined with gene expression and SNPs data separately, and then the three were combined. Using the gene expression and clinical data with CS, the accuracy ranged from 0.77 to 0.95, and the AUC ranged from 0.79 to 0.93. The highest performing model was the AdaBoost classifier with no feature selection (accuracy = 0.95 and AUC = 0.93, Figs. 1c, 2c). Using the SNPs and clinical data with cognitive score, the highest performing models were the Random Forest and the AdaBoost classifiers with no feature selection at an AUC of 0.93 and an accuracy of 0.94 (Figs. 1f, 2f). Using the combination of SNPs, gene expression, and clinical features as input, the AdaBoost classifier shows the best performance with LASSO feature selection method used (AUC of 0.94 and accuracy of 0.95, Figs. 1i, 2i).

Overall, the best performance was observed using all three gene, SNPs and clinical data with CS as input. Detailed results for all models, feature selection methods, and input types are presented in Supplementary Table S1. However, this paper focuses specifically on the utilization of blood biomarkers (genes and SNPs), our subsequent analyses will focus on the best performing models within these two categories for further model interpretation and feature analysis. Specifically, we will closely examine the top-performing gene-based model (Random Forest Classifier with Chi-squre feature selection) and the most top-performing SNP-based model (Adaboost classifier with no feature selection). Table  2 provides a performance comparison of our three best-performing models with previously published models. Prior studies often emphasized on either high AUC or accuracy, but not both, indicating a potential limitation. Hence, we are such to report both AUC and accuracy metrics. Including Mild Cognitive Impairment (MCI) participants is crucial for early detection, a factor often overlooked in prior research. Our SNP-based model outperformed a previous deep learning model^36^. Furthermore, our gene-based model demonstrated performance on par with the best-performing prior model, while also exhibiting enhanced sensitivity for early detection on ADNI data^22^.

**Table 2:** Performance comparison with previously published methods.

Model	Classification	Inputs	Accuracy	AUC	Reference
Random Forest with Chi-Square feature selection	Binary (CN, MCI/AD)	Gene expression and clinical data (no CS)	0.65	0.65	This work
AdaBoost model with no feature selection	Binary (CN, MCI/AD)	SNPs and clinical data (no CS)	0.67	0.63	This work
SVM model with MI feature selection	Binary (CN, MCI/AD)	SNPs and gene and clinical (with CS)	0.95	0.94	This work
Deep neural network (DNN)	Binary (CN, AD)	Blood gene expression	NA	0.656	Lee and Lee^22^
SVM	Binary (CN, AD)	Blood gene expression	NA	0.620	Lee and Lee^22^
BSWiMS-LASSO-RPART ensemble	Binary (CN, AD)	SNPs	0.677	0.719	Oriol et al.^25^
Deep learning models (DL)	Binary (CN, MCI/AD)	SNPs	0.66	NA	Venugopalan et al.^36^

Evaluation datasets were derived from ADNI by the respective authors.

BSWiMS: bootstrap stage-wise model selection; LASSO: least absolute shrinkage and selection operator; RPART: recursive partitioning and regression trees.

### Model explanation with SHAP

**Figure 3:**
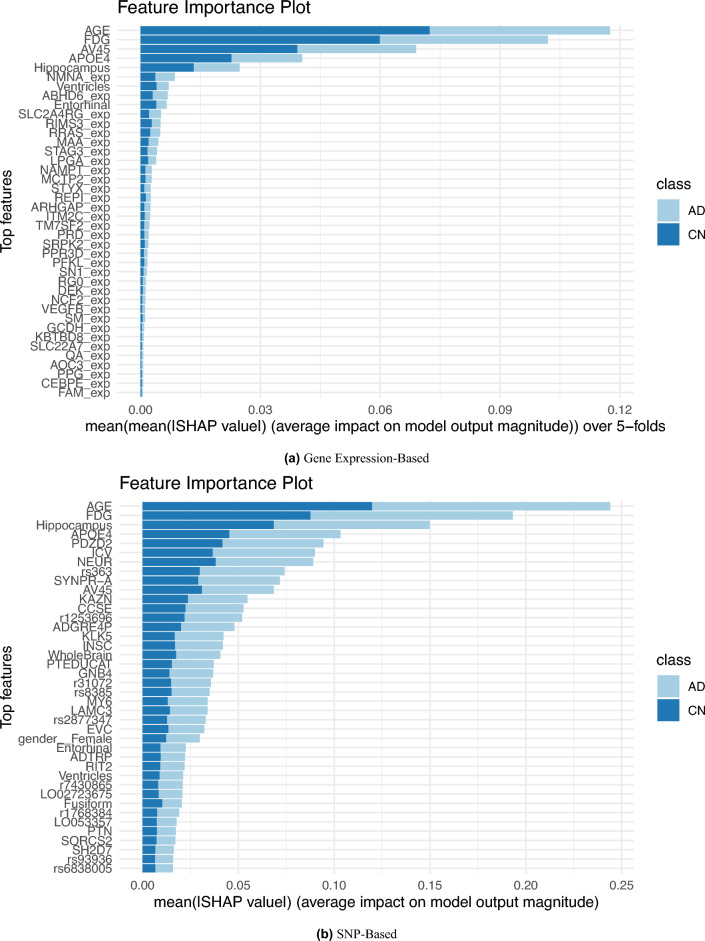
SHAP feature importance results showing top 40 most influential features for the prediction of (**a**) gene expression-based model, and (**b**) SNP-based model.

SHAP provides a framework for quantifying each feature’s contribution to model predictions, where each feature is given a value (SHAP scores), which are derived from cooperative game theory, to indicate its influence on the model’s output.The SHAP scores were calculated and their absolute values for the best-performing blood-biomarker based models: the gene-based Random Forest with Chi-square feature selection using gene expression and clinical data without CS (accuracy = 0.65, AUC = 0.65) and the SNP-based AdaBoost model with no feature selection using SNPs and clinical data without CS (accuracy = 0.67, AUC = 0.63). The top SHAP scores are summarized for the top 40 selected features in Fig. 3a and b. The larger the absolute value of the SHAP value, the more influential the feature is in making a prediction. Interestingly, most of the features prioritized by SHAP were blood biomarker features, with certain clinical features consistently ranking among the top. For both CN and MCI/AD prediction, the “AGE” has been marked as the most influential feature, with SHAP importance values greater than 0.65. Additional important clinical features for MCI/AD prediction include the imaging based FDG ((Fluorodeoxyglucose)) and AV45 (Florbetapir) which respectively measure glucose metabolism in the brain and detect beta-amyloid plaques in the brain. APOE4 is a specific variant of the apolipoprotein E (APOE) gene which is considered a major genetic risk factor for late-onset Alzheimer’s disease (AD). Its existence is shown as an important feature in the predictions.

### SHAP-prioritized genetic features

In addition to clinical features, certain genetic features (gene expression and SNPs) are also among the top SHAP-selected features influencing model prediction. From the overall feature importance plot (Fig.  3), NMNAT1, ABHD6, and SLC2A4RG gene loci have been prioritized in the gene expression-based model, while PDZD2, NEURL1, and SYNPR-A have been prioritized in the SNP-based model. These genes have been linked to brain aging and neurodegenerative processes making them promising leads for novel markers of AD pathophysiology and lend credibility to our model. A brief literature review was performed for the selected genes and the findings have been summarized below.

From the selected genes, NMNAT1 (Nicotinamide Mononucleotide Adenylyltransferase 1) has been suggested to play a pivotal role is safeguarding axons from degeneration^37,38^. It is a crucial enzyme in cellular metabolism, facilitating the production of nicotinamide adenine dinucleotide (NAD+), an essential coenzyme involved in various cellular processes. In one study, researchers observed that increasing the expression of NMNAT1 may have the potential to reverse neuronal degeneration^39^. This effect is believed to be achieved through its involvement in regulating oxidative stress and inhibiting cell death. The study by Marrs^40^ in 2010 identified ABHD6 as a significant serine hydrolase involved in the degradation of 2-arachidonoyl glycerol in the nervous system. This degradation is a crucial mechanism for regulating the levels of 2-AG in the synaptic cleft, which is important for maintaining proper neuronal communication and overall neurological function. ABHD6 has been further confirmed to be present in the mature human hippocampus^41^, suggesting potential distinct contributions from both neurons and glial cells to its overall levels in Alzheimer’s disease brains. In one study, the potential therapeutic benefits of ABHD6 inactivation in demyelinating condition have been demonstrated by inhibition of ABHD6^42^. Notably, SLC2A4RG was identified as one of the key genes influencing the hippocampus in Alzheimer’s disease^43^. SLC2A4RG is a transcriptional activator shuttling between nucleus and cytoplasm^44^, and is suggested to play an important role in the etiology of brain disease like glioblastoma and may be a potential therapeutic target^45^.

Interesting, PDZ-domain containing-2 (PDZD2) which has been identified as a novel protein detectable in both the fetal pancreas and our isolated pancreatic progenitor cells (PPCs) since early research was deemed as a significant feature^46^. It promotes proliferation of fetal pancreatic cells without them turning into specialized islet cells^47^. These findings hold promise for refining techniques in islet transplantation therapy, a key approach in treating diabetes. Although PDZD2 is mostly implicated in pancreatic development, it is important to highlight that diabetes is an established risk factor of AD and further study on PDZD2 could uncover correlations with Alzheimer’s disease. NEURL1, Neuralized1, which is an E3 ubiquitin ligase^48^ has been associated with learning and memory difficulties when it is downregulated^49^. SYNPR has been identified as the top marker genes for inhibitory neurons^50^. The National Center for Biotechnology Information (NCBI) predicts that it will be found as an essential membrane component in synaptic vesicles and neuron projections^51^. SNYNPR’s potential involvement in memory recognition, as demonstrated in a 2022 study^52^, coupled with its role in neurotransmitter modulation, provides insights into how imbalances may contribute to cognitive impairment, suggesting it could be a viable therapeutic target if its significance in the pathophysiology of AD is further confirmed. Additionally, prioritized features included KAZN and RIMS3 proteins have been associated with severe AD^53,54^.

### Stratified case studies with SHAP

**Figure 4:**
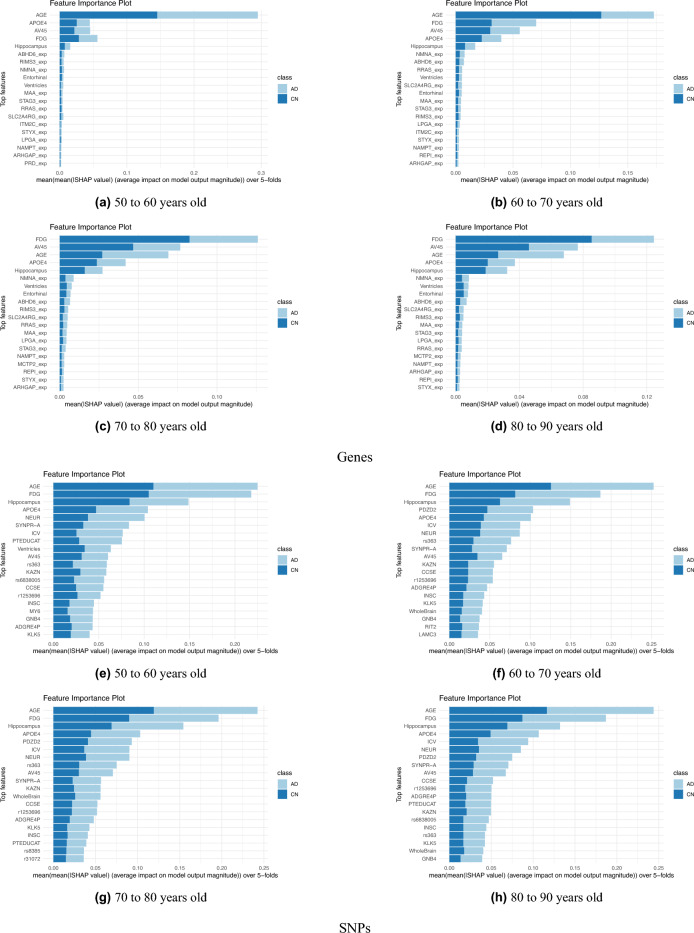
Feature importance plots for four stratified age groups for each of (**a**–**d**) gene expression-based model and (**e**–**h**) SNP-based model.

After observing that Age was the top feature in both the gene expression-based model and the SNP-based model according to mean absolute SHAP values, we were motivated to further explore its implications.The cohort was stratified into age groups with a span of 10 years for each group such that there was 4 groups: 50 to 60 years, 60 to 70 years, 70 to 80 years old, and 80 to 90 years old. The detailed cohort distribution by age is in Table 3 where 620 out of 623 participants were used as three of them are above 90 years old. As shown in Fig. 4 , the dynamic impact of Age on the models predictions across various age ranges is observed as its influence on distinguishing between CN and MCI/AD varies with advancing age. This feature importance plots provide the mean absolute SHAP values of the top 20 most influential features for each age group.

For the gene expression-based model, we can see that age holds the greatest influence in the first two age groups and specially for the 50–60 years old age group (Fig. 4a). This is because AD manifests differently in early-onset cases (occurring before 65) as opposed to late-onset cases (65 years and older) which makes age a more pronounced indicator in younger populations^55^. Also, the effect of other features on the models prediction increases as age group increases as can be seen going from Fig. 4b to c and lastly to 4d.

In the SNP-based model, Age emerged as the predominant feature of importance across all four age groups (Fig. 4e–h), with most other features maintaining a consistent ranking in terms of significance. In general, SNPs indicate genetic predisposition to diseases and can provide information about long-term disease risk. Gene expression, on the other hand, reflects the current activity of genes and can change over time, offering insights into disease progression and potential therapeutic targets. Both SNPs and gene expression are important tools in understanding and managing diseases, but they serve different roles in the diagnostic and prognostic process. The SNP-based model shows more uniformity in features selected and robustness across age groups, ensuring that the reliance on Age as a key predictor remains stable across different cohorts.

The insights obtained from this stratified examination extend to potential age-related biomarkers associated with the risk of Alzheimer’s disease (AD), such as the reduced expression of NMNAT1. This understanding improves the clinical interpretability of the model, allowing healthcare professionals to recognize the significance of Age in forecasting the risk of Alzheimer’s disease (AD) and facilitating educated conversations with patients regarding individualized healthcare choices.

**Table 3:** Stratified participants count in the four age groups.

Age group	CN	MCI/AD
50–60 years old	1	19
60–70 years old	30	125
70–80 years old	146	191
80–90 years old	35	73

## Discussion

The objective of this study was to create an interpretable machine learning (ML) classifier that enhances the precision of identifying cases of mild cognitive impairment (MCI)/Alzheimer’s disease (AD) and cognitively normal (CN) individuals using both gene expression and SNP data, surpassing the performance of previously published models. Additionally, the study aimed to highlight crucial gene features that play a significant role in the diagnosis of AD. The two gene models, gene-expression based and SNP-based, with the best performance were the Random Forest classifier with Chi-square feature selection and the AdaBoost classifier with no feature selection respectively. In the gene expression-based model, the input features from gene expression and clinical data without CS were processed using chi-square which assesses the independence between a feature and the target (ie. accurate prediction of MCI/AD cases) by comparing observed and expected frequencies in a contingency table, helping to identify statistically significant features. SHapley Additive exPlanations were used with both models to elucidate potential biomarkers for AD diagnosis from blood genotyping and clinical data with CS.

From the overall SHAP analysis, clinical features such as Age, FDG, AV45, and hippocampus were found to have high importance in the model’s predictions. This is expected since, with the absence of neuropsychological test scores, imaging features represent structural features of the AD brain. Early changes in FDG imaging suggest it may be able to predict which individuals among CN or MCI are most likely to progress to AD^56^, supported by studies on hypometabolism in AD-affected regions^57^. Moreover, smaller hippocampal volumes have been correlated with worse study partner-reported everyday cognition scores in another cohort^58^.

The genetic features identified by SHAP effectively encompass various aspects of early-stage Alzheimer’s disease. This significance is further highlighted by the decision not to incorporate clinical cognitive scores, as their inclusion might overshadow other crucial features. Axonal degenration safeguarding by the regulation of NMNAT1 protein, and synaptic memberane integrity indicated by ABHD, both being precursors of neurodegeneration. The identification of PDZD2 and NEURL1 genes, which have been linked to cognitive impairments related to learning and memory, serves as supportive evidence for the efficacy of the models employed.

Our models have demonstrated superior performance compared to previously published machine learning models, particularly due to their effectiveness in handling early-stage Mild Cognitive Impairment (MCI) participants, who were not considered in previous studies. The gene expression-based model (AUC = 0.65, accuracy = 0.65) is comparable to the Lee and Lee^22^ model (AUC = 0.656, accuracy = N/A). The SNP-based model, while displaying lower evaluation values, compensates for this by taking into account the additional MCI cohort, with (AUC = 0.63, accuracy = 0.67) it is comparable to the Oriol^25^ model (AUC = 0.719, accuracy = 0.67). Moreover, the identification of the most influential clinical and genetic features using SHAP demonstrates the utility and validity of our ML model. Nonetheless, we recognize the possible limitations of our study. In our model, we have utilized a predefined list of top gene features from another related study. The incorporation of the full SNPs and gen expression data may also prove useful in our model. Furthermore, our model is developed on a dataset of 623 patients from ADNI. We hope to refine and test our model with additional, independently curated external datasets besides ADNI to validate our model. Finally, we recognize that the potential biomarkers identified by our method would require experimental validation. In the future, we hope to refine our model with external validation datasets as mentioned previously. We also hope to incorporate other modes of input data in our workflow, such as non-coding SNPs. Epigenetic data may prove beneficial to the workflow, as evidence of epigenetic changes has been observed in PBMCs (peripheral blood mononuclear cells) of AD patients^59^. The incorporation of additional data may improve model performance further and disclose additional biomarkers for diagnosis and treatment.

From our study, we have identified potential gene loci associated with the degeneration of myelin and malfunction of synaptic vesicle membrane that indicate the early stages of neurodegeneration. Furthermore, the age group-stratified studies facilitated by SHAP offer more detailed insights from both clinical and genetic data tailored to specific age groups. Hence, our methodology, which omits the reliance on clinical cognitive scores, marks a significant stride towards uncovering novel genetic features associated with dementia. It is also hoped that our ML and SHAP workflow will help dispel black-box notions among clinicians and accelerate the adoption of machine learning in assisting clinical diagnosis.

## Methods

### Alzheimer’s Disease Neuroimaging Initiative (ADNI)

Data used in this article were obtained from the Alzheimer’s Disease Neuroimaging Initiative (ADNI) database (http://adni.loni.usc.edu). ADNI is a public-private partnership in 2003, led by Principal Investigator Michael W. Weiner, MD with the main goal of testing whether serial magnetic resonance imaging (MRI), positron emission tomography (PET), other biological markers, and clinical and neuropsychological assessment could track the progression of mild cognitive impairment (MCI) and early Alzheimer’s disease (AD). For up-to-date information, please see http://www.adni-info.org. In addition to MRI and PET neuroimaging of patients at regular intervals, ADNI has collected and analyzed whole blood samples for genotyping and gene expression analysis. Table 4 summarizes the genotyping data provided by ADNI. Blood gene expression profiling was conducted using Affymetrix Human Genome U219 Array for 744 patients in the ADNI2/ADNI-GO phase^24^. For this study, the authors utilised all the participants for whom both genetic and gene expression data were available in ADNI (623 participants). The selected cohort comprises 212 participants with a baseline diagnosis of cognitively normal (CN) and 411 participants with a diagnosis of Mild Cognitive Impairment (MCI) or Alzheimer’s Disease (AD), grouped together.

**Table 4:** ADNI genotyping data summary.

Phase	Platform	Variants	Genome assembly	DbSNP build
ADNI1	Illumina Human 610-Quad BeadChip	620901 SNP and CNV markers	hg18	129
ADNIGO/ADNI2	Illumina Human OmniExpress BeadChip	730525 SNP and CNV markers	hg18	129

The ADNI clinical data included patient demographics, brain functioning scores, neuropsychological test scores, and MRI volume measurements. Demographic information such as age, gender, ethnicity, education, and marital status have been included. The APOE4 variable indicates the presence of the APOE-$\epsilon 4$ allele, a known AD risk factor. PET measures for brain function include variables such as fluorodeoxyglucose (FDG), Pittsburgh compound B (PIB), and Florbetapir (AV45). Amyloid-$\beta$, tau and p-tau levels in cerebrospinal fluid (CSF) are indicated by the ABETA, TAU, and PTAU variables. The clinical dementia rating sum of boxes (CDRSB) variable provides the sum of all cognition and function scores from the Clinical Dementia Rating test. ADAS and MOCA are neuropsychological test variables used to assess cognitive capacity. The Mini-Mental State Exam (MMSE) variable reflects disease progression and cognitive changes over time. Rey’s Auditory Verbal Learning Test (RAVLT) variable is a neuropsychological test to examine episodic memory. Logical Memory-Delayed Recall Total Number of Story Units Recalled (LDELTOTAL) is another variable from neuropsychological tests that assesses an individual’s ability to remember information after some time. TRABSCOR variable denotes the time required to complete neuropsychological tests. Functional Activities Questionnaire (FAQ) assesses an individual’s reliance on others to perform daily life activities. Everyday cognitive evaluations (Ecog) are questionnaires used to assess the patient’s ability to perform daily tasks. The hippocampus, intracranial volume (ICV), Mid Temporal, Fusiform, Ventricles, Entorhinal, and Whole Brain are structural MRI variables. The Modified Preclinical Alzheimer Cognitive Composite (mPACC) variable assesses cognition, episodic memory, and time needed to complete tasks.

### Data preprocessing

Among the participants, 623 unique individuals randomly provided whole blood samples for gene expression assays at specific time points. Consequently, we selected these 623 patients and concurrently extracted their clinical data at the time of whole blood sample collection. Upon evaluation, the clinical data revealed that the majority of variables were not missing for most individuals or, at most, a small number of them. The ADNI dataset had the PIB and DIGISCOR variables missing for over 90% of individuals and were therefore removed. Around 35% of the selected participants do not have CSF biomarker variables (ABETA, TAU, PTAU). MRI variables are missing in 17% of the individuals, and FDG with AV45 variables are missing in 16% of the individuals. Therefore, missing data were identified and imputed with Multivariate Imputation By Chained Equations (MICE) using scikit-learn package in Python^60^. Imputation was performed on the training data and then applied to the test data. Supplementary Fig. S1 shows all variables and the number of missing values in each variable.

For performance purposes, the genotyping data utilized in this study comprises the top 121 markers reported in a previous 2023 study^61^. Exact feature list can be found in Supplementary Fig. S2. As reported, the “bim,” “bed,” and “fam” files are the three files that make up the original plink file format of the dataset. Subject characteristics are documented in the “fam” file. The location, name, and allele representation of SNPs (features) are kept in the “bim” file. Lastly, “bed” files provide machine codes that are unintelligible to humans. These codes are composed of 8-bit codes that map the data between fam and bim files and represent the genotype codes^61^.

### Machine learning pipeline

**Figure 5:**
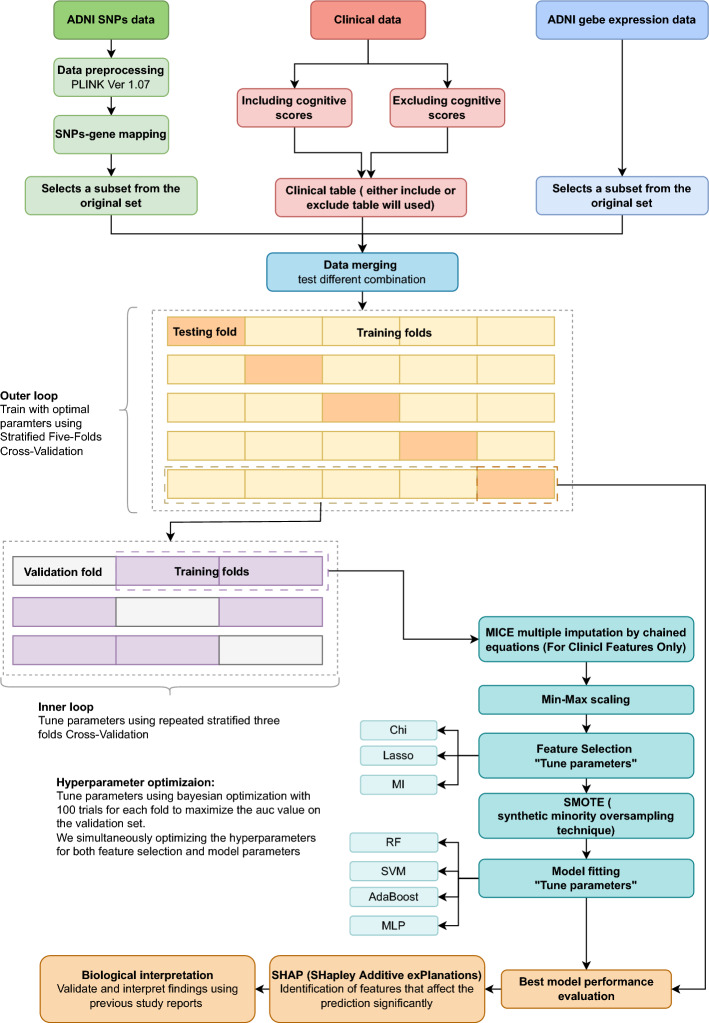
Machine learning pipeline for systematic model development.

The machine learning model development workflow is depicted in Fig. 5. The scikit-learn package in Python programming language v3.9.12 was used to develop the models. Further details of the workflow are discussed in the following sections.

### Preparation of input data

Different combinations of clinical data, gene expression, and SNP data were integrated and utilized to train the model as can be seen in Fig. 5. This training process involved a two-step cross-validation approach: first, a stratified five-fold cross-validation was conducted in the outer loop, followed by an additional stratified three-fold cross-validation in the inner loop. Target stratification was used in both the outer and inner loops to maintain the proportions of MCI/AD and CN participants. During the inner loop, MICE imputation is performed on the “training data”, Min-Max scaling is then applied before transforming the test dataset. Our dataset contains twice as many MCI/AD as CN participants (411 versus 212 cases) and is therefore unbalanced. This was addressed via utilizing the Synthetic Minority Oversampling Technique (SMOTE).

Four types of input data (SNPs, gene expression, clinical data, and clinical data without cognitive scores) were used separately and combined to create single modal and multi-modal data inputs for the machine learning models. Multi-modal inputs include paired combinations of the four inputs, as well as the combination of all four together. The features in multi-modal inputs were merged before feature selection.

### Feature selection

In this study, three different feature selection methods were carried out on both single-model and multi-modal inputs, namely Chi-square, mutual information (MI), and Least Absolute Shrinkage and Selection Operator (LASSO). Additionally, a parallel analysis was conducted, wherein no feature selection was implemented. Before the selection process, there were a total of 45 clinical features, 2,702,858 SNPs, and 19,403 gene expression features available. From the SNPs and gene expression features, 121 top features were chosen based on a well-established and previously published study^61^ enhancing the robustness of our genetic data. The number of features selected by each method varies as it is an optimized hyperarameter. The features selected by each method and feature selection method are provided in Supplementary Data 1.

### Classification models and hyperparameter optimization

To binary classify the subjects as either CN or MCI/AD, Support Vector Machine (SVM), Random Forest (RF), AdaBoost (AB), and Multi-Layer Perceptron classifier (MLP) models were implemented. Bayesian optimization with five-fold cross-validation and repeated stratified three-fold Cross-Validation was used to fine-tune the hyperparameters with 100 trials for each fold to maximize the auc value on the validation set. We simultaneously optimizing the hyperparameters for both feature selection and model parameters. For the SVM model, the cost (C), gamma ($\gamma$), kernel, and class weight hyperparameters were optimized such that the cost ranged between 0.1 and 1000, and gamma values between 0.0001 and 0.001. The choice of the kernel was among linear, polynomial, radial basis function (RBF), or sigmoid and class weight was set to either balanced or none.

For the RF model, the maximum tree depth, maximum number of features, split quality criterion, and class weight were optimized. The maximum tree depth values were set at 3, 5, 7, and none, maximum feature numbers included the square root and log (base 2) of the number of features, as well as the total number of features. The criterion was Gini impurity, logistic loss, and entropy. Lastly, the class weight was set to be balanced, balanced subsample, and none.

For AB, the number of estimators, learning rate, and algorithm were optimized. The number of estimators was set at 50, 100, and 200. The learning rate was set at 0.1, 0.01, and 0.001. For the algorithm, we utilized Stagewise Additive Modeling using a Multi-class Exponential loss function (SAMME) and SAMME.R (which outputs class probabilities instead of discrete values 0 or 1).

For the MLP classifier, the hidden layer sizes, activation function, weight optimizer, L2 regularization strength, and learning rate were optimized. The hidden layers depth ranged from 10 to 100 with an increment of 10. The weight optimization algorithm choices included adaptive moment estimation(Adam), stochastic gradient descent (SGD), and Limited-memory Broyden-Fletcher-Goldfarb-Shanno (LBFGS). The learning rate was either set as a constant or specified according to adaptive or inverse scaling methods. The L2 strength values were set as 0.01, 0.001, or 0.0001.

The selected hyperparameters for each dataset and the extracted features are shown in Supplementary Data 2.

### Model performance metrics

Model performance is evaluated using the receiver-operator characteristic curve (ROC-AUC) and accuracy. The formulae for accuracy is shown below:

1 $$\begin{aligned} Accuracy = \frac{TP + TN}{TP + TN + FP + FN} \end{aligned}$$

Here, the number of correctly predicted MCI/AD cases are True positive (TP), and CN participants incorrectly predicted as MCI/AD are assigned False Positive (FP). Correctly predicted CN participants are True Negative (TN), while MCI/AD participants wrongly predicted as CN are assigned False Negative (FN).

### SHAP model interpretation

Lundberg and Lee^35^ have proposed SHapley Additive exPlanations (SHAP) technique to explain model predictions, which computes a unified measure of feature importance using game theory. To calculate SHAP values, each feature’s contribution to the predicted value is estimated by comparing predictions over different combinations of features. The SHAP value for a feature is the average of all the marginal contributions to predictions from all possible feature combinations. SHAP values indicate the magnitude of difference that each feature makes to the final predicted value, starting from a base expected value. SHAP values were computed for the best-performing model to identify features that have the highest impact on model performance and are therefore potential biomarkers for prodromal and advanced Alzheimer’s. Genes were extracted from the most influential SHAP features, and a comprehensive review of the existing literature was conducted to establish connections between our findings and experimental evidence.

### Stratified case studies with SHAP

To examine the insights provided by SHAP, the cohort was stratified by age in 10 years interval from 50 to 90 years old. This stratified examination also provides nuanced insights into whether Age assumes a more prominent role in discriminating between the two classes within specific age brackets, potentially signifying a stronger association with AD development or progression in late adulthood. A feature importance plot was generated for each selected age-group, allowing for a comprehensive understanding of the impact of each feature on the final prediction outcome.

### Statistical analysis

Differences in clinical features between CN and MCI/AD participants were analyzed using statistical tests for significance. The t-test was used for parametric continuous variables (with equal variance assumption), while the Mann-Whitney U test was used for non-parametric continuous variables. The Chi-square $(\chi ^2)$ test was used to test categorical variables hypotheses (with continuity correction), while Fisher’s exact test was used for smaller sample sizes (small cell counts). All statistical tests were performed at the 95% significance level.

### Supplementary Information

Supplementary Information 1.

Supplementary Information 2.

Supplementary Information 3.

## Data Availability

The dataset analysed in this study is publicly available in the Alzheimer’s Disease Neuroimaging Initiative (ADNI) repository, (http://adni.loni.usc.edu) (Accession Number: sa000002).
