# Author Correction: Predicting early Alzheimer’s with blood biomarkers and clinical features

**DOI:** 10.1038/s41598-024-71400-8

**Published:** 2024-08-30

**Authors:** Muaath Ebrahim AlMansoori, Sherlyn Jemimah, Ferial Abuhantash, Aamna AlShehhi

**Affiliations:** 1 Department of Biomedical Engineering, Khalifa University, P.O. Box: 127788, Abu Dhabi, United Arab Emirates; 2 Healthcare Engineering Innovation Center (HEIC), Khalifa University, P.O. Box: 127788, Abu Dhabi, United Arab Emirates

Correction to: *Scientific Reports* 10.1038/s41598-024-56489-1, published online 13 March 2024

The original version of this Article contained an error in Reference 49, which was incorrectly given as:

“Akın, S., Gültekin, F. & Güler, E. M. The relationship of learning and memory disfunction with neurl1 and rgs14 genes in patients with autism spectrum disorders. Acta Medica Alanya 6, 207–213 (2022).”

The correct reference is listed below:

“Eciroglu, H., Şener, E. F., Öztop, D. B., Özmen, S., Kaan, D., & Özkul, Y. (2022). The relationship of learning and memory disfunction with neurl1 and rgs14 genes in patients with autism spectrum disorders. Acta Medica Alanya, 6, 207–213. DOI: 10.30565/medalanya.1136820.”

The original Article has been corrected.

