# Supplementary material for: Predicting early Alzheimer’s with blood biomarkers and clinical features
Source: Sci Rep. 2024 Mar 13;14:6039. doi: 10.1038/s41598-024-56489-1 (PMC10933308; doi:10.1038/s41598-024-56489-1)
Supplement: Supplementary file 3 — Supplementary Information 3. [file 41598_2024_56489_MOESM3_ESM.pdf]

Predicting Early Alzheimer's with Blood Biomarkers and Clinical Features

Supplementary Material

Muaath Ebrahim AlMansoori[1,+], Sherlyn Jemimah[1,+], Ferial Abuhantash[1], Aamna AlShehhi[1,2*] and the Alzheimer's Disease Neuroimaging Initiative[†]

[1] Department of Biomedical Engineering, Khalifa University, P.O. Box: 127788, Abu Dhabi, United Arab Emirates

[2] Healthcare Engineering Innovation Center (HEIC), Khalifa University, P.O. Box: 127788, Abu Dhabi, United Arab Emirates

[*] aamna.alshehhi@ku.ac.ae

[+] these authors contributed equally to this work

[†] Data used in the preparation of this article were obtained from the Alzheimer's Disease Neuroimaging Initiative (ADNI) database (adni.loni.usc.edu). As such, the investigators within ADNI contributed to the design and implementation of ADNI and/or provided data but did not participate in the analysis or writing of this report. A complete listing of ADNI investigators can be found at

Supplementary Table S1: Full Results

The table below summarizes the full accuracy and AUC results for all the model runs using all the different combinations of data. Where "Clinical" refers to clinical data without cognitive scores.

Data Used	Model	FeatureSelection	Accuracy	AUC
Clinical with cognitive + Genes	AdaBoost	Chi	0.950258	0.943959
Clinical with cognitive + Genes	AdaBoost	Lasso	0.955097	0.943012
Clinical with cognitive + Genes	AdaBoost	MI	0.955097	0.946441
Clinical with cognitive + Genes	AdaBoost	None	0.959897	0.948994
Clinical with cognitive + Genes	MLP	Chi	0.845948	0.844246
Clinical with cognitive + Genes	MLP	Lasso	0.83791	0.845497
Clinical with cognitive + Genes	MLP	MI	0.918168	0.918411
Clinical with cognitive + Genes	MLP	None	0.847471	0.83662
Clinical with cognitive + Genes	RandomForest	Chi	0.914877	0.916024
Clinical with cognitive + Genes	RandomForest	Lasso	0.942206	0.937906
Clinical with cognitive + Genes	RandomForest	MI	0.95991	0.947958
Clinical with cognitive + Genes	RandomForest	None	0.95991	0.947958

Clinical with cognitive + Genes	SVM	Chi	0.932606	0.93634
Clinical with cognitive + Genes	SVM	Lasso	0.834503	0.84742
Clinical with cognitive + Genes	SVM	MI	0.951858	0.946244
Clinical with cognitive + Genes	SVM	None	0.844297	0.848933
Clinical with cognitive + SNPs	AdaBoost	Chi	0.942258	0.933385
Clinical with cognitive + SNPs	AdaBoost	Lasso	0.942245	0.934407
Clinical with cognitive + SNPs	AdaBoost	MI	0.945445	0.935725
Clinical with cognitive + SNPs	AdaBoost	None	0.956684	0.948782
Clinical with cognitive + SNPs	MLP	Chi	0.829884	0.832287
Clinical with cognitive + SNPs	MLP	Lasso	0.863445	0.872821
Clinical with cognitive + SNPs	MLP	MI	0.913342	0.914907
Clinical with cognitive + SNPs	MLP	None	0.816981	0.812535
Clinical with cognitive + SNPs	RandomForest	Chi	0.942245	0.933271
Clinical with cognitive + SNPs	RandomForest	Lasso	0.955084	0.943138
Clinical with cognitive + SNPs	RandomForest	MI	0.950284	0.942853
Clinical with cognitive + SNPs	RandomForest	None	0.95991	0.947958
Clinical with cognitive + SNPs	SVM	Chi	0.935794	0.938765
Clinical with cognitive + SNPs	SVM	Lasso	0.940581	0.941288
Clinical with cognitive + SNPs	SVM	MI	0.947071	0.944964
Clinical with cognitive + SNPs	SVM	None	0.783174	0.795139
Clinical + Genes + SNPs	AdaBoost	Chi	0.683729	0.650279
Clinical + Genes + SNPs	AdaBoost	Lasso	0.67249	0.628345
Clinical + Genes + SNPs	AdaBoost	MI	0.667677	0.652265
Clinical + Genes + SNPs	AdaBoost	None	0.648439	0.624763
Clinical + Genes + SNPs	MLP	Chi	0.624387	0.594981
Clinical + Genes + SNPs	MLP	Lasso	0.606748	0.577253
Clinical + Genes + SNPs	MLP	MI	0.653355	0.658338
Clinical + Genes + SNPs	MLP	None	0.64049	0.60172
Clinical + Genes + SNPs	RandomForest	Chi	0.6564	0.62522
Clinical + Genes + SNPs	RandomForest	Lasso	0.654865	0.633759
Clinical + Genes + SNPs	RandomForest	MI	0.624348	0.645674
Clinical + Genes + SNPs	RandomForest	None	0.6532	0.637646
Clinical + Genes + SNPs	SVM	Chi	0.662942	0.637192
Clinical + Genes + SNPs	SVM	Lasso	0.658194	0.607164
Clinical + Genes + SNPs	SVM	MI	0.640413	0.630077
Clinical + Genes + SNPs	SVM	None	0.650142	0.604498
Clinical + Genes	AdaBoost	Chi	0.677316	0.674083
Clinical + Genes	AdaBoost	Lasso	0.650194	0.644439
Clinical + Genes	AdaBoost	MI	0.635535	0.624157
Clinical + Genes	AdaBoost	None	0.637135	0.632596
Clinical + Genes	MLP	Chi	0.60031	0.639176
Clinical + Genes	MLP	Lasso	0.616297	0.62735
Clinical + Genes	MLP	MI	0.617768	0.6441

Clinical + Genes	MLP	None	0.617948	0.622579
Clinical + Genes	RandomForest	Chi	0.691794	0.683009
Clinical + Genes	RandomForest	Lasso	0.664503	0.664651
Clinical + Genes	RandomForest	MI	0.643755	0.652019
Clinical + Genes	RandomForest	None	0.630774	0.641349
Clinical + Genes	SVM	Chi	0.627523	0.650277
Clinical + Genes	SVM	Lasso	0.634039	0.640105
Clinical + Genes	SVM	MI	0.638748	0.647315
Clinical + Genes	SVM	None	0.619613	0.626784
Clinical + SNPs	AdaBoost	Chi	0.688503	0.66344
Clinical + SNPs	AdaBoost	Lasso	0.646955	0.622899
Clinical + SNPs	AdaBoost	MI	0.669484	0.638578
Clinical + SNPs	AdaBoost	None	0.693497	0.663721
Clinical + SNPs	MLP	Chi	0.602065	0.572975
Clinical + SNPs	MLP	Lasso	0.640452	0.607081
Clinical + SNPs	MLP	MI	0.589123	0.557235
Clinical + SNPs	MLP	None	0.601845	0.570317
Clinical + SNPs	RandomForest	Chi	0.653329	0.643306
Clinical + SNPs	RandomForest	Lasso	0.645265	0.635089
Clinical + SNPs	RandomForest	MI	0.649987	0.644289
Clinical + SNPs	RandomForest	None	0.690258	0.667096
Clinical + SNPs	SVM	Chi	0.65169	0.603675
Clinical + SNPs	SVM	Lasso	0.656568	0.641749
Clinical + SNPs	SVM	MI	0.643703	0.628109
Clinical + SNPs	SVM	None	0.658065	0.599296
Clinical with cognitive	AdaBoost	Chi	0.945406	0.940374
Clinical with cognitive	AdaBoost	Lasso	0.945432	0.942767
Clinical with cognitive	AdaBoost	MI	0.950258	0.94522
Clinical with cognitive	AdaBoost	None	0.940632	0.934503
Clinical with cognitive	MLP	Chi	0.844297	0.848933
Clinical with cognitive	MLP	Lasso	0.890994	0.890165
Clinical with cognitive	MLP	MI	0.868452	0.87398
Clinical with cognitive	MLP	None	0.846013	0.85493
Clinical with cognitive	RandomForest	Chi	0.900516	0.897195
Clinical with cognitive	RandomForest	Lasso	0.929406	0.92472
Clinical with cognitive	RandomForest	MI	0.955058	0.946567
Clinical with cognitive	RandomForest	None	0.903729	0.898546
Clinical with cognitive	SVM	Chi	0.929458	0.93382
Clinical with cognitive	SVM	Lasso	0.940671	0.936771
Clinical with cognitive	SVM	MI	0.932632	0.930662
Clinical with cognitive	SVM	None	0.911781	0.909182
Clinical	AdaBoost	Chi	0.561948	0.576179
Clinical	AdaBoost	Lasso	0.666194	0.66257

Clinical	AdaBoost	MI	0.646955	0.654791
Clinical	AdaBoost	None	0.674245	0.666356
Clinical	MLP	Chi	0.603639	0.619582
Clinical	MLP	Lasso	0.617987	0.640807
Clinical	MLP	MI	0.5716	0.586319
Clinical	MLP	None	0.632606	0.650857
Clinical	RandomForest	Chi	0.589174	0.602872
Clinical	RandomForest	Lasso	0.634103	0.640444
Clinical	RandomForest	MI	0.642129	0.639415
Clinical	RandomForest	None	0.654981	0.660976
Clinical	SVM	Chi	0.578077	0.621885
Clinical	SVM	Lasso	0.632555	0.657818
Clinical	SVM	MI	0.639032	0.65582
Clinical	SVM	None	0.651871	0.681481
Genes + SNPs	AdaBoost	Chi	0.646942	0.600761
Genes + SNPs	AdaBoost	Lasso	0.60991	0.571437
Genes + SNPs	AdaBoost	MI	0.585832	0.550087
Genes + SNPs	AdaBoost	None	0.585832	0.536073
Genes + SNPs	MLP	Chi	0.578103	0.563588
Genes + SNPs	MLP	Lasso	0.58751	0.551694
Genes + SNPs	MLP	MI	0.553703	0.539781
Genes + SNPs	MLP	None	0.585794	0.55525
Genes + SNPs	RandomForest	Chi	0.58591	0.534226
Genes + SNPs	RandomForest	Lasso	0.577794	0.542932
Genes + SNPs	RandomForest	MI	0.566581	0.552863
Genes + SNPs	RandomForest	None	0.595535	0.550233
Genes + SNPs	SVM	Chi	0.63409	0.574144
Genes + SNPs	SVM	Lasso	0.613252	0.563076
Genes + SNPs	SVM	MI	0.626116	0.552045
Genes + SNPs	SVM	None	0.630852	0.561725
Genes	AdaBoost	Chi	0.547239	0.514765
Genes	AdaBoost	Lasso	0.524955	0.487213
Genes	AdaBoost	MI	0.556865	0.540848
Genes	AdaBoost	None	0.561755	0.518049
Genes	MLP	Chi	0.519923	0.529723
Genes	MLP	Lasso	0.526606	0.525446
Genes	MLP	MI	0.539458	0.514163
Genes	MLP	None	0.502271	0.496181
Genes	RandomForest	Chi	0.540916	0.52963
Genes	RandomForest	Lasso	0.528077	0.516504
Genes	RandomForest	MI	0.519948	0.513047
Genes	RandomForest	None	0.518516	0.514825
Genes	SVM	Chi	0.548826	0.537992

Genes	SVM	Lasso	0.560142	0.534955
Genes	SVM	MI	0.590723	0.52304
Genes	SVM	None	0.569742	0.544488
SNPs	AdaBoost	Chi	0.613187	0.56752
SNPs	AdaBoost	Lasso	0.605187	0.556972
SNPs	AdaBoost	MI	0.643639	0.597685
SNPs	AdaBoost	None	0.613355	0.562284
SNPs	MLP	Chi	0.571471	0.550913
SNPs	MLP	Lasso	0.571445	0.555445
SNPs	MLP	MI	0.536026	0.553876
SNPs	MLP	None	0.577948	0.554586
SNPs	RandomForest	Chi	0.597252	0.55966
SNPs	RandomForest	Lasso	0.552	0.525953
SNPs	RandomForest	MI	0.577935	0.548285
SNPs	RandomForest	None	0.616439	0.557364
SNPs	SVM	Chi	0.624387	0.55549
SNPs	SVM	Lasso	0.619626	0.55626
SNPs	SVM	MI	0.635639	0.551207
SNPs	SVM	None	0.65809	0.567093

Supplementary Table S2: Characteristics terminology definition

Characteristic	Definition
FDG	Average FDG-PET of angular, temporal, and posterior cingulate
AV45	Reference region - florbetapir mean of whole cerebellum. Regions defined by Freesurfer; see Jagust lab PDF on LONI for details
ABETA	CSF amyloid beta
CDRSB	clinical dementia rating
ADAS11	Alzheimer's Disease Assessment Scale–Cognitive subscale 11 (ADAS-Cog)
ADAS13	Alzheimer's Disease Assessment Scale–Cognitive subscale 13 (ADAS-Cog)
ADASQ4	Q4: delayed word recall
MMSE	Mini-Mental State Examination
RAVLT_immediate	Rey Auditory Verbal Learning Test (RAVLT) Immediate (sum of 5 trials)
LDELTOTAL	Logical Memory - Delayed Recall
TRABSCOR	Trails B
FAQ	Functional Activities Questionnaires
MOCA	Montreal Cognitive Assessment
EcogPtMem	Everyday Cognition - Participant Self Report – memory
EcogPtLang	Language
EcogPtVisspat	Visual
EcogPtPlan	Plan
EcogPtOrgan	Organ
EcogPtDivatt	Div attention
EcogSPMem	Everyday Cognition – Study Partner Report
ICV	intracranial volume
mPACCdigit	ADNI modified Preclinical Alzheimer's Cognitive Composite (PACC) with Digit Symbol Substitution
mPACCtrailsB	ADNI modified Preclinical Alzheimer's Cognitive Composite (PACC) with Trails B

Supplementary Figure S1 - ***Showing all clinical variables and the number of missing values in each clinical variable.***

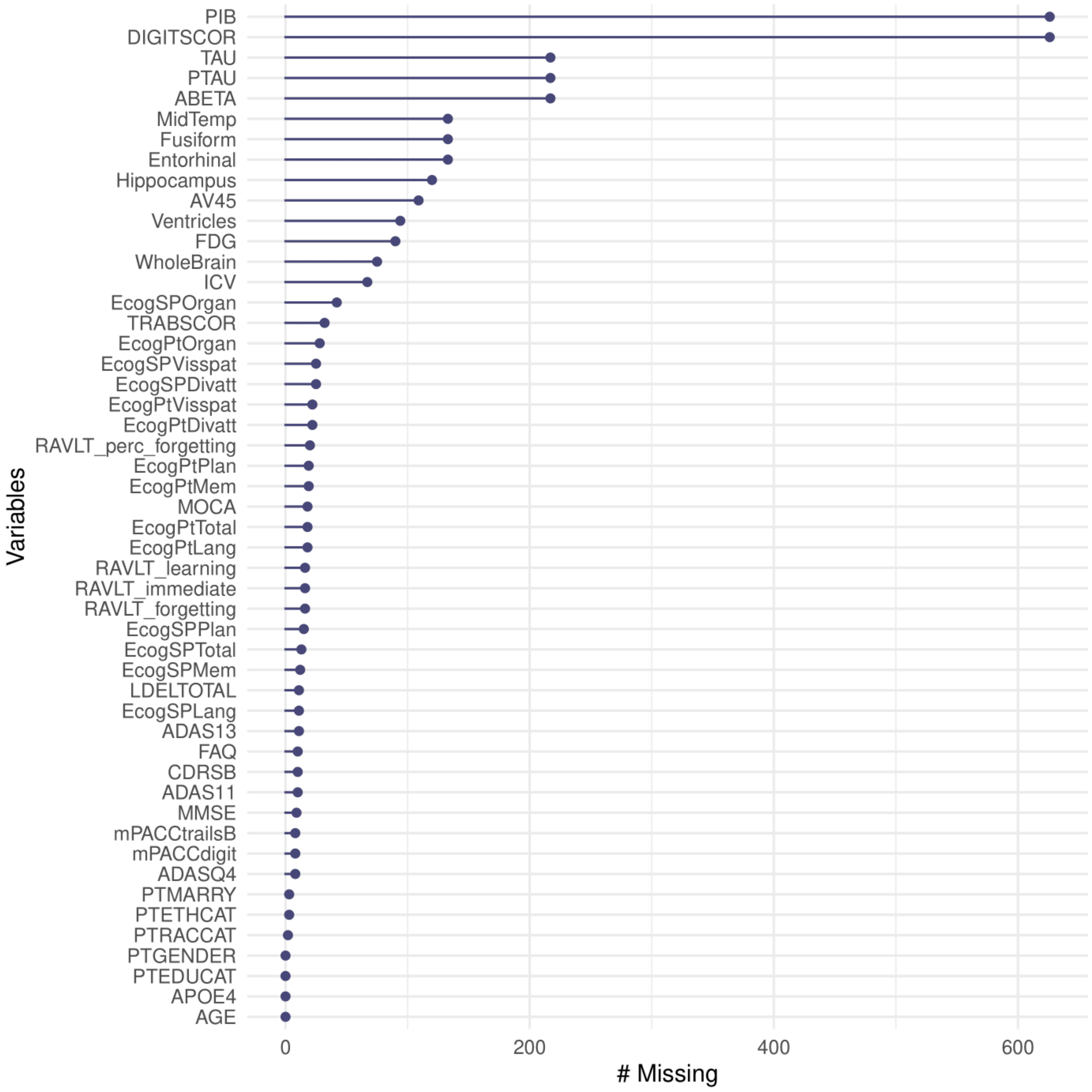

Supplementary Figure S2 - S*howing all gene features used.*

rs6116375_CC	rs10176603_TT	rs7747741_GG	rs4290760_CC	rs16864809_TT
rs2654986_TC	rs10031325_CC	rs701880_CC	rs11680332_GG	rs7679260_CC
rs11768384_GG	rs16889565_GA	rs9296691_TC	rs628482_GG	rs9389952_TT
rs2075650_AA	rs2877347_CC	rs4953672_CC	rs518385_TT	rs10804812_CC
rs7342676_CC	rs6114605_GA	rs10068900_GG	rs2577322_CC	rs618236_CC
rs4964453_TT	rs7618348_CC	rs2834714_TT	rs11869174_CT	rs1945624_AA
rs10790928_TT	rs9595108_CC	rs6838005_CC	rs11733633_AA	rs2577322_TT
rs2208322_AA	rs17068548_GG	rs10514486_CC	rs911892_TT	rs7807731_TT
rs7519796_AA	rs13211072_TT	rs7149949_TT	rs3812568_AA	rs2136613_TT
rs10222715_TT	rs6132022_TT	rs2725790_CT	rs799447_GG	rs344783_TT
rs10793982_TT	rs793291_AA	rs11655031_TT	rs17745021_CT	rs1495813_CC
rs775879_GG	rs3771389_CT	rs2833427_CC	rs13245564_GG	rs9410486_GG
rs4837137_AA	rs6695731_CC	rs8007000_TT	rs2305252_AA	rs7096762_AA
rs1789250_AA	rs10044783_CC	rs17430865_CT	rs4472075_AA	rs2309777_GG
rs4868468_AA	rs17345545_CC	rs3815360_CC	rs4793902_TT	rs9515168_GT
rs11752811_TT	rs871049_CC	rs17430865_TT	rs168825_GG	rs6569364_AA
rs2075650_GG	rs4953672_AA	rs11922179_AA	rs6838005_TC	rs12988856_TT
rs2697303_AA	rs2075650_GA	rs1186685_TT	rs775879_AA	rs1891265_GG
rs362584_AA	rs1479884_GG	rs7320494_AA	rs6903956_AA	
rs8000805_GG	rs11253696_AA	rs7206002_GG	rs12480224_AA	
rs10879839_TT	rs13135230_GG	rs367369_TT	rs2339298_TT	
rs2286343_AA	rs10888578_TT	rs1328179_TT	rs7413155_AC	
rs939720_CC	rs7999171_GG	rs4689705_TT	rs9595108_AC	
rs7165661_TT	rs12312628_CC	rs705904_CC	rs6929400_CC	
rs2867922_TT	rs10101666_TT	rs9381936_CC	rs268909_TT	

https://doi.org/10.1371/journal.pone.0283712.t003
